# Diethyl 1,8-diphenyl-11-oxatricyclo­[6.2.1.0^2,7^]undeca-2,4,6-triene-9,10-dicarboxyl­ate

**DOI:** 10.1107/S1600536813002791

**Published:** 2013-02-02

**Authors:** B. Balakrishnan, Meganathan Nandakumar, P. R. Seshadri, Arasambattu K. Mohanakrishnan

**Affiliations:** a Department of Physics, P. T. Lee Chengalvaraya Naicker College of Engineering & Technology, Kancheepuram 631 502, India; b Department of Organic Chemistry, University of Madras, Guindy Campus, Chennai 600 025, India; c PostGraduate & Research Department of Physics, Agurchand Manmull Jain College, Chennai 600 114, India

## Abstract

The title compound, C_28_H_26_O_5_, is the Diels–Alder adduct from 1,3-diphenyl­benzo[*c*]furan and diethyl maleate. The mol­ecule comprises of a fused tricyclic system containing two five-membered rings, which are in envelope conformations with the O atom at the flap, and a six-membered ring adopting a boat conformation. The dihedral angle between phenyl substituents in the 1,8-positions is 55.1 (1)°. The ethyl groups are disordered over two sets of sites, with occupancy ratios of 0.648 (9):0.352 (9) and 0.816 (1):0.184 (1). In the crystal, pairs of C—H⋯π inter­actions link the mol­ecules into inversion dimers.

## Related literature

For background to Diels–Alder reactions, see: Stevens & Richards (1997 ▶). For related structures, see: Doboszewski *et al.* (2010 ▶); Toze *et al.* (2011 ▶); Bailey *et al.* (1995 ▶); Ohwada *et al.* (2001 ▶); Takahashi *et al.* (2003 ▶). For puckering and asymmetry parameters, see: Cremer & Pople(1975 ▶); Nardelli (1983 ▶).
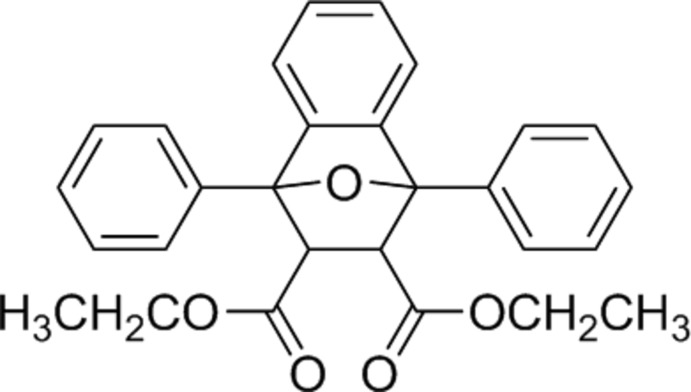


## Experimental

### 

#### Crystal data

C_28_H_26_O_5_
*M* *_r_* = 442.49
Triclinic, 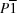

*a* = 9.7126 (3) Å
*b* = 11.5930 (3) Å
*c* = 12.5989 (5) Å
α = 115.013 (2)°
β = 107.126 (2)°
γ = 97.431 (1)°
*V* = 1174.60 (7) Å^3^
*Z* = 2
Mo *K*α radiation
μ = 0.09 mm^−1^
*T* = 293 K
0.30 × 0.20 × 0.20 mm

#### Data collection

Bruker Kappa APEXII CCD diffractometer
Absorption correction: multi-scan (*SADABS*; Bruker, 2004 ▶) *T*_min_ = 0.952, *T*_max_ = 0.991
20057 measured reflections
4124 independent reflections
3271 reflections with *I* > 2σ(*I*)
*R*_int_ = 0.031

#### Refinement

*R*[*F* ^2^ > 2σ(*F* ^2^)] = 0.041
*wR*(*F* ^2^) = 0.110
*S* = 1.06
4124 reflections
323 parameters
40 restraints
H-atom parameters constrained
Δρ_max_ = 0.23 e Å^−3^
Δρ_min_ = −0.18 e Å^−3^

### 

Data collection: *APEX2* (Bruker, 2004 ▶); cell refinement: *APEX2* and *SAINT* (Bruker, 2004 ▶); data reduction: *SAINT*; program(s) used to solve structure: *SIR92* (Altomare *et al.*, 1993 ▶); program(s) used to refine structure: *SHELXL97* (Sheldrick, 2008 ▶); molecular graphics: *ORTEP-3 for Windows* (Farrugia, 2012 ▶) and *PLATON* (Spek, 2009 ▶); software used to prepare material for publication: *PLATON* and *publCIF* (Westrip, 2010 ▶).

## Supplementary Material

Crystal structure: contains datablock(s) I, global. DOI: 10.1107/S1600536813002791/im2416sup1.cif

Structure factors: contains datablock(s) I. DOI: 10.1107/S1600536813002791/im2416Isup2.hkl

Supplementary material file. DOI: 10.1107/S1600536813002791/im2416Isup3.cml

Additional supplementary materials:  crystallographic information; 3D view; checkCIF report

## Figures and Tables

**Table 1:** Hydrogen-bond geometry (Å, °) *Cg*1 and *Cg*2 are the centroids of the C2–C7and C9–C14 rings respectively.

*D*—H⋯*A*	*D*—H	H⋯*A*	*D*⋯*A*	*D*—H⋯*A*
C28—H28*B*⋯*Cg*1^i^	0.98	3.00	3.601 (7)	121
C5—H5⋯*Cg*2^ii^	0.93	2.89	3.693 (3)	145

Symmetry codes: (i) 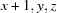; (ii) 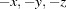.

**Fig. 1.**
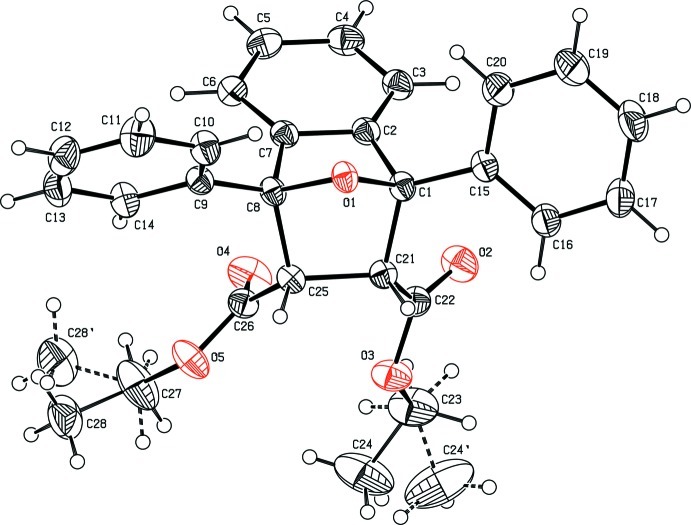
Molecular structure of the title compound, showing 30% probability displacement ellipsoids.

**Fig. 2.**
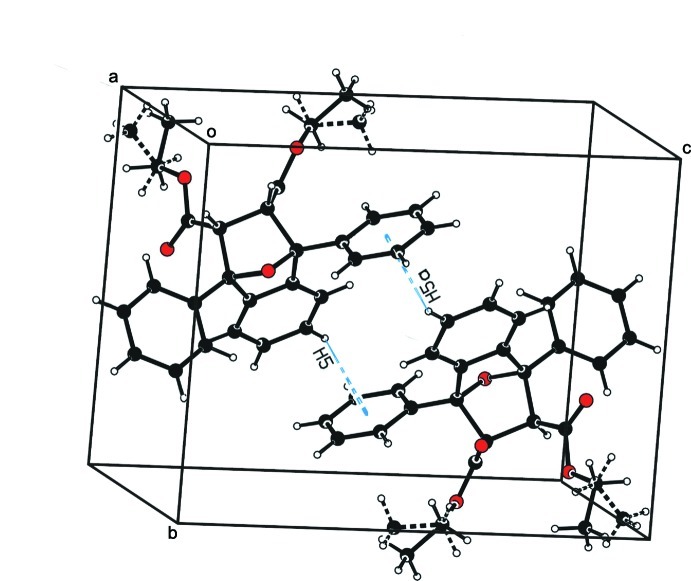
A view of the C—H···π interactions in the crystal structure of the title compound.

## supplementary crystallographic information

### Comment

Diels-Alder adducts from the reaction of anthracene with dienophiles have been used in a variety of applications, including the synthesis of discrete molecular architectures such as molecular gears (Stevens & Richards 1997). The title compound, C_28_H_26_O_5_, comprises a fused tricyclic system and two phenyl rings attached with this system (Fig. 1). The tricyclic system consists of two 5-membered rings and one aromatic ring. In addition, two ethyl carboxylate units are attached to the tricyclic system. Geometrical parameters agree well with reported structures (Doboszewski *et al.* 2010; Toze *et al.* 2011; Bailey *et al.* 1995; Ohwada *et al.* 2001; Takahashi *et al.* 2003). The five membered ring C_1_\C_2_\C_7_\C_8_\O_1_ adopts an envelope conformation with O_1_ displaced by -0.752 Å from the mean plane of the other ring atoms C_1_\C_2_\C_7_\C_8_. The puckering parameters (Cremer & Pople, 1975) and asymmetry parameters (Nardelli, 1983) are q_2_ = 0.511 (1) Å, φ = -36.5 (1)°, Δ_S_(O_1_) = 0.004 (1)° and Δ_2_(O_1_) = 0.302 (1)°. The second five membered ring C_1_\C_21_\C_25_\C_8_\O_1_ also adopts an envelope conformation with O_1_ displaced by -0.834) Å from the mean plane of the other ring atoms C_1_\ C_21_\C_25_\C_8_.The puckering parameters (Cremer & Pople, 1975) and asymmetry parameters (Nardelli, 1983) are q_2_ = 0.592 (1) Å, φ = 144.2 (1)°, Δ_S_(O_1_) = 0.002 (1)° and Δ_2_(O_1_) = 0.354 (1)°. The six membered ring C_1_/C_2_/C_7_/C_8_/C_25_/C_21_ adopts boat conformation with puckering parameter q_2_ = 0.951 (1) Å, θ=89.7 (1)° and φ = 180.8 (8)°.

The dihedral angle between the rings C_1_/C_2_/C_7_/C_8_/O_1_ and C_1_/C_21_/C_25_/C_8_/O_1_ is 66.7 (1)°. The dihedral angle between terminal phenyl rings is 55.1 (1)°. One of these aromatic substituents (C9 - C14) is almost orthogonal to the plane formed by the six atoms C1, C2, C7, C8, C25 and C21 of the tricyclic ring, the dihedral angle being 81.1 (1)° (Nardelli, 1983). Atoms C24 and C28 of the ester groups are disordered over two sites with occupancy ratios of 0.648 (9): 0.352 (9) and 0.816 (1): 0.184 (1). In the ester group, the C26—O5—C27—C28 torsion angle for major component is -155.4 (3)° and the C26—O5—C27—C28' torsion angle for minor component is -115 (1)°. The second ester group, C22—O3—C23—C24 connected to the tricyclic ring is almost co-planar as evidenced by torsion angle of -162 (3)°, while the C22—O3—C23—C24' is considerably twisted from the ring with a torsion angle of 137.7 (1)°.

Centrosymmetric dimers are formed by C—H···π (C5—H5···*Cg*2and C28—H28B···*Cg*1) interactions, where *Cg*1 and *Cg*2 are centroids of the C2—C7 and C9—C14 rings, respectively (Fig. 2).

### Experimental

1, 3-diphenylisobenzofuran (1.00 g, 2.26 mmole) was dissolved in toluene (25 ml) and treated with 2 equivalents of diethyl maleate (0.78 g, 4.52 mmole). The reaction mixture was refluxed and the reaction was monitored by TLC. After 8 h, the mixture was cooled to room temperature. The solvent was removed and the residue was purified by column chromatography (Silica gel, 10%, ethyl acetate/hexane) to give the adduct as a white solid. Yield: 1.42 g (87%). This adduct was crystallized from CHCl_3_/CH_3_OH (3:1) by slow evaporation of tzhe solvent.

### Refinement

All H atoms were positioned geometrically and allowed to ride on their parent atoms, with (C—H= 0.93–0.96 Å), and *U*_iso_(H) = 1.5 *U*_eq_(C) for methyl H atoms and 1.2 U_eq_(C) for other H atoms. The carbon atoms of ester groups are disordered over two sites with occupancy ratio of 0.648 (9): 0.352 (9) and 0.816 (1): 0.184 (1).

### Crystal data

**Table:**

C_28_H_26_O_5_	*Z* = 2
*M**_r_* = 442.49	*F*(000) = 468
Triclinic, *P*1	*D*_x_ = 1.251 Mg m^−^^3^
Hall symbol: -P 1	Mo *K*α radiation, λ = 0.71073 Å
*a* = 9.7126 (3) Å	Cell parameters from 7905 reflections
*b* = 11.5930 (3) Å	θ = 2.3–26.1°
*c* = 12.5989 (5) Å	µ = 0.09 mm^−^^1^
α = 115.013 (2)°	*T* = 293 K
β = 107.126 (2)°	Block, colourless
γ = 97.431 (1)°	0.30 × 0.20 × 0.20 mm
*V* = 1174.60 (7) Å^3^	

### Data collection

**Table:**

Bruker Kappa APEXII CCD diffractometer	4124 independent reflections
Radiation source: fine-focus sealed tube	3271 reflections with *I* > 2σ(*I*)
Graphite monochromator	*R*_int_ = 0.031
ω and φ scan	θ_max_ = 25.0°, θ_min_ = 2.0°
Absorption correction: multi-scan (*SADABS*; Bruker, 2004)	*h* = −11→11
*T*_min_ = 0.952, *T*_max_ = 0.991	*k* = −13→13
20057 measured reflections	*l* = −14→14

### Refinement

**Table:**

Refinement on *F*^2^	Secondary atom site location: difference Fourier map
Least-squares matrix: full	Hydrogen site location: inferred from neighbouring sites
*R*[*F*^2^ > 2σ(*F*^2^)] = 0.041	H-atom parameters constrained
*wR*(*F*^2^) = 0.110	*w* = 1/[σ^2^(*F*_o_^2^) + (0.0503*P*)^2^ + 0.2754*P*] where *P* = (*F*_o_^2^ + 2*F*_c_^2^)/3
*S* = 1.06	(Δ/σ)_max_ < 0.001
4124 reflections	Δρ_max_ = 0.23 e Å^−^^3^
323 parameters	Δρ_min_ = −0.18 e Å^−^^3^
40 restraints	Extinction correction: *SHELXL97* (Sheldrick, 2008), Fc^*^=kFc[1+0.001xFc^2^λ^3^/sin(2θ)]^-1/4^
Primary atom site location: structure-invariant direct methods	Extinction coefficient: 0.022 (2)

### Special details

**Table:**

Geometry. All e.s.d.'s (except the e.s.d. in the dihedral angle between two l.s. planes) are estimated using the full covariance matrix. The cell e.s.d.'s are taken into account individually in the estimation of e.s.d.'s in distances, angles and torsion angles; correlations between e.s.d.'s in cell parameters are only used when they are defined by crystal symmetry. An approximate (isotropic) treatment of cell e.s.d.'s is used for estimating e.s.d.'s involving l.s. planes.
Refinement. Refinement of *F*^2^ against ALL reflections. The weighted *R*-factor *wR* and goodness of fit *S* are based on *F*^2^, conventional *R*-factors *R* are based on *F*, with *F* set to zero for negative *F*^2^. The threshold expression of *F*^2^ > σ(*F*^2^) is used only for calculating *R*-factors(gt) *etc*. and is not relevant to the choice of reflections for refinement. *R*-factors based on *F*^2^ are statistically about twice as large as those based on *F*, and *R*- factors based on ALL data will be even larger.

### Fractional atomic coordinates and isotropic or equivalent isotropic displacement parameters (Å^2^)

**Table:**

	*x*	*y*	*z*	*U*_iso_*/*U*_eq_	Occ. (<1)
C1	0.25522 (17)	0.49311 (15)	0.32261 (14)	0.0371 (4)	
C2	0.15307 (17)	0.39619 (15)	0.18491 (15)	0.0379 (4)	
C3	0.04433 (18)	0.40701 (18)	0.09315 (16)	0.0459 (4)	
H3	0.0216	0.4869	0.1104	0.055*	
C4	−0.0298 (2)	0.29520 (19)	−0.02527 (17)	0.0528 (5)	
H4	−0.1048	0.2995	−0.0882	0.063*	
C5	0.0060 (2)	0.17731 (19)	−0.05146 (17)	0.0527 (5)	
H5	−0.0451	0.1036	−0.1319	0.063*	
C6	0.11658 (18)	0.16679 (17)	0.03995 (15)	0.0451 (4)	
H6	0.1415	0.0877	0.0220	0.054*	
C7	0.18838 (17)	0.27769 (16)	0.15830 (15)	0.0374 (4)	
C8	0.31206 (17)	0.30534 (15)	0.27998 (14)	0.0359 (4)	
C9	0.32640 (17)	0.18935 (16)	0.30214 (14)	0.0388 (4)	
C10	0.2801 (2)	0.17340 (18)	0.38992 (17)	0.0497 (4)	
H10	0.2416	0.2364	0.4375	0.060*	
C11	0.2905 (2)	0.0648 (2)	0.4078 (2)	0.0641 (5)	
H11	0.2598	0.0556	0.4678	0.077*	
C12	0.3456 (2)	−0.02946 (19)	0.33801 (19)	0.0629 (5)	
H12	0.3520	−0.1026	0.3503	0.075*	
C13	0.3913 (2)	−0.01565 (19)	0.25001 (19)	0.0595 (5)	
H13	0.4289	−0.0795	0.2024	0.071*	
C14	0.3818 (2)	0.09273 (18)	0.23183 (17)	0.0508 (4)	
H14	0.4129	0.1012	0.1717	0.061*	
C15	0.19656 (18)	0.59988 (16)	0.39805 (15)	0.0412 (4)	
C16	0.2768 (2)	0.73281 (17)	0.46900 (17)	0.0501 (4)	
H16	0.3726	0.7605	0.4716	0.060*	
C17	0.2160 (2)	0.8256 (2)	0.5366 (2)	0.0619 (5)	
H17	0.2709	0.9152	0.5840	0.074*	
C18	0.0751 (3)	0.7860 (2)	0.5339 (2)	0.0694 (6)	
H18	0.0337	0.8486	0.5781	0.083*	
C19	−0.0040 (3)	0.6539 (2)	0.4657 (2)	0.0808 (7)	
H19	−0.0986	0.6264	0.4651	0.097*	
C20	0.0557 (2)	0.5613 (2)	0.3978 (2)	0.0675 (6)	
H20	0.0006	0.4718	0.3513	0.081*	
C21	0.41920 (17)	0.53186 (15)	0.33086 (14)	0.0373 (4)	
H21	0.4822	0.5948	0.4203	0.045*	
C22	0.44240 (19)	0.59954 (17)	0.25567 (16)	0.0437 (4)	
C23	0.6189 (3)	0.6761 (3)	0.1870 (3)	0.0843 (7)	
H23A	0.5378	0.6334	0.1037	0.101*	0.648 (9)
H23B	0.6296	0.7707	0.2272	0.101*	0.648 (9)
H23C	0.6275	0.6053	0.1147	0.101*	0.352 (9)
H23D	0.5344	0.7046	0.1542	0.101*	0.352 (9)
C24	0.7558 (5)	0.6543 (9)	0.1749 (7)	0.111 (2)	0.648 (9)
H24A	0.7854	0.6986	0.1322	0.167*	0.648 (9)
H24B	0.7410	0.5605	0.1264	0.167*	0.648 (9)
H24C	0.8332	0.6891	0.2577	0.167*	0.648 (9)
C24'	0.7516 (14)	0.7734 (15)	0.2455 (11)	0.145 (5)	0.352 (9)
H24D	0.8317	0.7420	0.2786	0.217*	0.352 (9)
H24E	0.7470	0.8502	0.3139	0.217*	0.352 (9)
H24F	0.7701	0.7969	0.1854	0.217*	0.352 (9)
C25	0.46133 (17)	0.39910 (15)	0.29990 (14)	0.0369 (4)	
H25	0.5433	0.4121	0.3753	0.044*	
C26	0.50768 (18)	0.34621 (17)	0.18808 (16)	0.0429 (4)	
C27	0.6848 (3)	0.2517 (3)	0.1148 (3)	0.0946 (9)	
H27A	0.5988	0.1986	0.0346	0.113*	0.816 (11)
H27B	0.7439	0.3214	0.1091	0.113*	0.816 (11)
H27C	0.7864	0.3029	0.1384	0.113*	0.184 (11)
H27D	0.6191	0.2482	0.0380	0.113*	0.184 (11)
C28	0.7750 (7)	0.1691 (5)	0.1376 (5)	0.0845 (14)	0.816 (11)
H28A	0.8599	0.2218	0.2170	0.127*	0.816 (11)
H28B	0.8100	0.1316	0.0701	0.127*	0.816 (11)
H28C	0.7155	0.0987	0.1407	0.127*	0.816 (11)
C28'	0.674 (4)	0.1155 (18)	0.091 (2)	0.107 (6)	0.184 (11)
H28D	0.7263	0.1167	0.1692	0.160*	0.184 (11)
H28E	0.7190	0.0746	0.0314	0.160*	0.184 (11)
H28F	0.5701	0.0657	0.0564	0.160*	0.184 (11)
O1	0.27065 (12)	0.40156 (10)	0.37414 (10)	0.0381 (3)	
O2	0.34976 (15)	0.63584 (14)	0.20159 (14)	0.0626 (4)	
O3	0.58390 (14)	0.62090 (14)	0.26383 (12)	0.0578 (4)	
O4	0.44061 (15)	0.33349 (16)	0.08622 (12)	0.0669 (4)	
O5	0.63261 (15)	0.31127 (15)	0.21722 (13)	0.0639 (4)	

### Atomic displacement parameters (Å^2^)

**Table:**

	*U*^11^	*U*^22^	*U*^33^	*U*^12^	*U*^13^	*U*^23^
C1	0.0423 (9)	0.0387 (9)	0.0422 (8)	0.0167 (7)	0.0217 (7)	0.0250 (7)
C2	0.0369 (8)	0.0413 (9)	0.0449 (9)	0.0135 (7)	0.0208 (7)	0.0251 (8)
C3	0.0432 (9)	0.0509 (10)	0.0566 (10)	0.0182 (8)	0.0207 (8)	0.0351 (9)
C4	0.0433 (10)	0.0649 (12)	0.0511 (10)	0.0126 (9)	0.0111 (8)	0.0348 (10)
C5	0.0478 (10)	0.0534 (11)	0.0453 (10)	0.0065 (9)	0.0114 (8)	0.0207 (9)
C6	0.0441 (9)	0.0432 (9)	0.0467 (9)	0.0123 (8)	0.0180 (8)	0.0206 (8)
C7	0.0362 (8)	0.0420 (9)	0.0426 (9)	0.0136 (7)	0.0204 (7)	0.0240 (7)
C8	0.0400 (8)	0.0402 (9)	0.0364 (8)	0.0177 (7)	0.0209 (7)	0.0205 (7)
C9	0.0379 (8)	0.0416 (9)	0.0405 (8)	0.0150 (7)	0.0149 (7)	0.0223 (7)
C10	0.0592 (11)	0.0519 (10)	0.0551 (10)	0.0238 (9)	0.0306 (9)	0.0330 (9)
C11	0.0857 (15)	0.0619 (12)	0.0657 (12)	0.0253 (11)	0.0354 (11)	0.0441 (11)
C12	0.0762 (13)	0.0505 (11)	0.0665 (12)	0.0234 (10)	0.0171 (11)	0.0380 (10)
C13	0.0658 (12)	0.0504 (11)	0.0646 (12)	0.0306 (10)	0.0231 (10)	0.0275 (10)
C14	0.0591 (11)	0.0512 (11)	0.0554 (10)	0.0265 (9)	0.0296 (9)	0.0296 (9)
C15	0.0471 (9)	0.0437 (9)	0.0453 (9)	0.0213 (8)	0.0246 (8)	0.0257 (8)
C16	0.0540 (10)	0.0470 (10)	0.0577 (11)	0.0209 (9)	0.0282 (9)	0.0266 (9)
C17	0.0754 (14)	0.0457 (11)	0.0687 (12)	0.0270 (10)	0.0345 (11)	0.0243 (10)
C18	0.0797 (15)	0.0655 (14)	0.0784 (14)	0.0430 (12)	0.0491 (12)	0.0300 (12)
C19	0.0700 (14)	0.0721 (16)	0.1089 (19)	0.0301 (12)	0.0609 (14)	0.0317 (14)
C20	0.0611 (12)	0.0514 (11)	0.0899 (15)	0.0183 (10)	0.0472 (12)	0.0220 (11)
C21	0.0398 (8)	0.0398 (9)	0.0367 (8)	0.0132 (7)	0.0180 (7)	0.0198 (7)
C22	0.0458 (9)	0.0444 (9)	0.0457 (9)	0.0126 (8)	0.0213 (8)	0.0238 (8)
C23	0.0781 (15)	0.116 (2)	0.0984 (17)	0.0188 (15)	0.0470 (14)	0.0806 (17)
C24	0.078 (3)	0.199 (6)	0.141 (5)	0.057 (4)	0.072 (3)	0.130 (5)
C24'	0.156 (8)	0.143 (9)	0.114 (7)	−0.044 (7)	0.041 (6)	0.077 (6)
C25	0.0383 (8)	0.0445 (9)	0.0346 (8)	0.0160 (7)	0.0170 (7)	0.0219 (7)
C26	0.0429 (9)	0.0476 (10)	0.0441 (9)	0.0144 (8)	0.0231 (8)	0.0229 (8)
C27	0.1091 (19)	0.134 (2)	0.1008 (18)	0.0792 (19)	0.0855 (17)	0.0674 (18)
C28	0.096 (3)	0.078 (3)	0.109 (3)	0.046 (2)	0.073 (3)	0.044 (2)
C28'	0.121 (11)	0.099 (10)	0.109 (10)	0.047 (9)	0.091 (8)	0.024 (8)
O1	0.0466 (6)	0.0410 (6)	0.0411 (6)	0.0207 (5)	0.0259 (5)	0.0243 (5)
O2	0.0622 (8)	0.0750 (9)	0.0825 (9)	0.0300 (7)	0.0334 (7)	0.0593 (8)
O3	0.0490 (7)	0.0785 (9)	0.0669 (8)	0.0152 (6)	0.0296 (6)	0.0497 (7)
O4	0.0635 (8)	0.1043 (11)	0.0431 (7)	0.0297 (8)	0.0281 (6)	0.0382 (7)
O5	0.0624 (8)	0.0935 (10)	0.0671 (8)	0.0468 (8)	0.0445 (7)	0.0463 (8)

### Geometric parameters (Å, º)

**Table:**

C1—O1	1.4618 (18)	C20—H20	0.9300
C1—C15	1.506 (2)	C21—C22	1.507 (2)
C1—C2	1.519 (2)	C21—C25	1.561 (2)
C1—C21	1.558 (2)	C21—H21	0.9800
C2—C3	1.380 (2)	C22—O2	1.196 (2)
C2—C7	1.385 (2)	C22—O3	1.330 (2)
C3—C4	1.384 (3)	C23—C24'	1.356 (9)
C3—H3	0.9300	C23—C24	1.425 (5)
C4—C5	1.381 (3)	C23—O3	1.456 (2)
C4—H4	0.9300	C23—H23A	0.9700
C5—C6	1.385 (2)	C23—H23B	0.9700
C5—H5	0.9300	C23—H23C	0.9700
C6—C7	1.377 (2)	C23—H23D	0.9700
C6—H6	0.9300	C24—H23C	1.1599
C7—C8	1.515 (2)	C24—H24A	0.9600
C8—O1	1.4462 (17)	C24—H24B	0.9600
C8—C9	1.499 (2)	C24—H24C	0.9600
C8—C25	1.579 (2)	C24'—H24D	0.9600
C9—C10	1.380 (2)	C24'—H24E	0.9600
C9—C14	1.388 (2)	C24'—H24F	0.9600
C10—C11	1.380 (3)	C25—C26	1.509 (2)
C10—H10	0.9300	C25—H25	0.9800
C11—C12	1.367 (3)	C26—O4	1.190 (2)
C11—H11	0.9300	C26—O5	1.327 (2)
C12—C13	1.369 (3)	C27—C28	1.439 (4)
C12—H12	0.9300	C27—O5	1.456 (2)
C13—C14	1.379 (3)	C27—C28'	1.460 (15)
C13—H13	0.9300	C27—H27A	0.9700
C14—H14	0.9300	C27—H27B	0.9700
C15—C16	1.377 (2)	C27—H27C	0.9700
C15—C20	1.382 (3)	C27—H27D	0.9700
C16—C17	1.385 (3)	C28—H27C	1.5364
C16—H16	0.9300	C28—H28A	0.9600
C17—C18	1.372 (3)	C28—H28B	0.9600
C17—H17	0.9300	C28—H28C	0.9600
C18—C19	1.367 (3)	C28'—H28D	0.9600
C18—H18	0.9300	C28'—H28E	0.9600
C19—C20	1.377 (3)	C28'—H28F	0.9600
C19—H19	0.9300		
			
O1—C1—C15	109.38 (12)	C24'—C23—C24	55.8 (7)
O1—C1—C2	100.40 (11)	C24'—C23—O3	115.4 (5)
C15—C1—C2	117.40 (13)	C24—C23—O3	108.9 (3)
O1—C1—C21	98.75 (11)	C24'—C23—H23A	134.7
C15—C1—C21	119.01 (13)	C24—C23—H23A	109.9
C2—C1—C21	108.56 (12)	O3—C23—H23A	109.9
C3—C2—C7	120.90 (15)	C24'—C23—H23B	55.3
C3—C2—C1	133.41 (14)	C24—C23—H23B	109.9
C7—C2—C1	105.69 (13)	O3—C23—H23B	109.9
C2—C3—C4	117.89 (16)	H23A—C23—H23B	108.3
C2—C3—H3	121.1	C24'—C23—H23C	105.3
C4—C3—H3	121.1	C24—C23—H23C	54.0
C5—C4—C3	121.02 (16)	O3—C23—H23C	108.1
C5—C4—H4	119.5	H23A—C23—H23C	59.5
C3—C4—H4	119.5	H23B—C23—H23C	141.9
C4—C5—C6	121.17 (17)	C24'—C23—H23D	112.1
C4—C5—H5	119.4	C24—C23—H23D	142.3
C6—C5—H5	119.4	O3—C23—H23D	108.2
C7—C6—C5	117.62 (16)	H23A—C23—H23D	49.7
C7—C6—H6	121.2	H23B—C23—H23D	62.3
C5—C6—H6	121.2	H23C—C23—H23D	107.4
C6—C7—C2	121.39 (15)	C23—C24—H23C	42.6
C6—C7—C8	133.22 (15)	C23—C24—H24A	109.5
C2—C7—C8	105.38 (13)	H23C—C24—H24A	102.3
O1—C8—C9	111.29 (12)	C23—C24—H24B	109.5
O1—C8—C7	100.90 (11)	H23C—C24—H24B	73.2
C9—C8—C7	117.23 (13)	C23—C24—H24C	109.5
O1—C8—C25	99.22 (11)	H23C—C24—H24C	144.4
C9—C8—C25	116.92 (12)	C23—C24'—H24D	109.5
C7—C8—C25	108.62 (12)	C23—C24'—H24E	109.5
C10—C9—C14	118.20 (15)	H24D—C24'—H24E	109.5
C10—C9—C8	120.89 (14)	C23—C24'—H24F	109.5
C14—C9—C8	120.87 (14)	H24D—C24'—H24F	109.5
C9—C10—C11	120.56 (17)	H24E—C24'—H24F	109.5
C9—C10—H10	119.7	C26—C25—C21	115.19 (13)
C11—C10—H10	119.7	C26—C25—C8	113.88 (13)
C12—C11—C10	120.61 (18)	C21—C25—C8	100.93 (11)
C12—C11—H11	119.7	C26—C25—H25	108.8
C10—C11—H11	119.7	C21—C25—H25	108.8
C13—C12—C11	119.64 (17)	C8—C25—H25	108.8
C13—C12—H12	120.2	O4—C26—O5	123.90 (15)
C11—C12—H12	120.2	O4—C26—C25	125.44 (15)
C12—C13—C14	120.18 (18)	O5—C26—C25	110.63 (14)
C12—C13—H13	119.9	C28—C27—O5	110.3 (2)
C14—C13—H13	119.9	C28—C27—C28'	37.9 (11)
C13—C14—C9	120.82 (17)	O5—C27—C28'	105.7 (7)
C13—C14—H14	119.6	C28—C27—H27A	109.6
C9—C14—H14	119.6	O5—C27—H27A	109.6
C16—C15—C20	118.52 (16)	C28'—C27—H27A	76.6
C16—C15—C1	123.58 (15)	C28—C27—H27B	109.6
C20—C15—C1	117.89 (15)	O5—C27—H27B	109.6
C15—C16—C17	120.46 (17)	C28'—C27—H27B	139.8
C15—C16—H16	119.8	H27A—C27—H27B	108.1
C17—C16—H16	119.8	C28—C27—H27C	76.5
C18—C17—C16	120.32 (19)	O5—C27—H27C	110.6
C18—C17—H17	119.8	C28'—C27—H27C	112.8
C16—C17—H17	119.8	H27A—C27—H27C	133.7
C19—C18—C17	119.53 (18)	H27B—C27—H27C	35.6
C19—C18—H18	120.2	C28—C27—H27D	133.2
C17—C18—H18	120.2	O5—C27—H27D	110.6
C18—C19—C20	120.3 (2)	C28'—C27—H27D	108.4
C18—C19—H19	119.8	H27A—C27—H27D	33.7
C20—C19—H19	119.8	H27B—C27—H27D	76.5
C19—C20—C15	120.8 (2)	H27C—C27—H27D	108.7
C19—C20—H20	119.6	C27—C28—H27C	37.9
C15—C20—H20	119.6	C27—C28—H28A	109.5
C22—C21—C1	115.77 (13)	C27—C28—H28B	109.5
C22—C21—C25	117.58 (13)	C27—C28—H28C	109.5
C1—C21—C25	102.46 (12)	C27—C28'—H28D	109.5
C22—C21—H21	106.8	C27—C28'—H28E	109.5
C1—C21—H21	106.8	C27—C28'—H28F	109.5
C25—C21—H21	106.8	C8—O1—C1	98.16 (10)
O2—C22—O3	124.14 (15)	C22—O3—C23	116.23 (15)
O2—C22—C21	125.26 (15)	C26—O5—C27	116.02 (16)
O3—C22—C21	110.49 (14)		
			
O1—C1—C2—C3	149.26 (17)	C15—C16—C17—C18	0.2 (3)
C15—C1—C2—C3	30.9 (2)	C16—C17—C18—C19	1.1 (3)
C21—C1—C2—C3	−107.74 (19)	C17—C18—C19—C20	−1.4 (4)
O1—C1—C2—C7	−31.15 (14)	C18—C19—C20—C15	0.5 (4)
C15—C1—C2—C7	−149.53 (13)	C16—C15—C20—C19	0.8 (3)
C21—C1—C2—C7	71.84 (15)	C1—C15—C20—C19	179.5 (2)
C7—C2—C3—C4	1.1 (2)	O1—C1—C21—C22	164.98 (12)
C1—C2—C3—C4	−179.40 (16)	C15—C1—C21—C22	−77.03 (18)
C2—C3—C4—C5	−1.2 (3)	C2—C1—C21—C22	60.83 (17)
C3—C4—C5—C6	0.3 (3)	O1—C1—C21—C25	35.69 (13)
C4—C5—C6—C7	0.7 (3)	C15—C1—C21—C25	153.68 (13)
C5—C6—C7—C2	−0.9 (2)	C2—C1—C21—C25	−68.46 (14)
C5—C6—C7—C8	−179.86 (16)	C1—C21—C22—O2	7.3 (2)
C3—C2—C7—C6	0.0 (2)	C25—C21—C22—O2	128.85 (18)
C1—C2—C7—C6	−179.70 (14)	C1—C21—C22—O3	−176.40 (13)
C3—C2—C7—C8	179.20 (14)	C25—C21—C22—O3	−54.89 (19)
C1—C2—C7—C8	−0.45 (15)	C22—C21—C25—C26	−5.5 (2)
C6—C7—C8—O1	−148.54 (17)	C1—C21—C25—C26	122.65 (14)
C2—C7—C8—O1	32.35 (15)	C22—C21—C25—C8	−128.65 (14)
C6—C7—C8—C9	−27.5 (2)	C1—C21—C25—C8	−0.49 (13)
C2—C7—C8—C9	153.35 (13)	O1—C8—C25—C26	−159.38 (12)
C6—C7—C8—C25	107.75 (19)	C9—C8—C25—C26	80.95 (17)
C2—C7—C8—C25	−71.36 (14)	C7—C8—C25—C26	−54.49 (16)
O1—C8—C9—C10	9.6 (2)	O1—C8—C25—C21	−35.34 (13)
C7—C8—C9—C10	−105.79 (18)	C9—C8—C25—C21	−155.02 (13)
C25—C8—C9—C10	122.60 (16)	C7—C8—C25—C21	69.54 (14)
O1—C8—C9—C14	−172.61 (14)	C21—C25—C26—O4	−49.0 (2)
C7—C8—C9—C14	72.00 (19)	C8—C25—C26—O4	67.0 (2)
C25—C8—C9—C14	−59.6 (2)	C21—C25—C26—O5	132.97 (15)
C14—C9—C10—C11	0.7 (3)	C8—C25—C26—O5	−111.08 (15)
C8—C9—C10—C11	178.53 (17)	C9—C8—O1—C1	−176.08 (12)
C9—C10—C11—C12	−0.6 (3)	C7—C8—O1—C1	−50.97 (13)
C10—C11—C12—C13	0.2 (3)	C25—C8—O1—C1	60.17 (12)
C11—C12—C13—C14	0.0 (3)	C15—C1—O1—C8	174.57 (12)
C12—C13—C14—C9	0.1 (3)	C2—C1—O1—C8	50.46 (12)
C10—C9—C14—C13	−0.5 (3)	C21—C1—O1—C8	−60.38 (12)
C8—C9—C14—C13	−178.31 (16)	O2—C22—O3—C23	−8.5 (3)
O1—C1—C15—C16	116.36 (17)	C21—C22—O3—C23	175.18 (17)
C2—C1—C15—C16	−130.19 (17)	C24'—C23—O3—C22	137.7 (10)
C21—C1—C15—C16	4.0 (2)	C24—C23—O3—C22	−162.0 (4)
O1—C1—C15—C20	−62.2 (2)	O4—C26—O5—C27	−1.3 (3)
C2—C1—C15—C20	51.2 (2)	C25—C26—O5—C27	176.78 (18)
C21—C1—C15—C20	−174.54 (16)	C28—C27—O5—C26	−155.4 (3)
C20—C15—C16—C17	−1.2 (3)	C28'—C27—O5—C26	−115.7 (14)
C1—C15—C16—C17	−179.73 (16)		

### Hydrogen-bond geometry (Å, º)

Cg1 and Cg2 are the centroids of the C2–C7and C9–C14 rings respectively.

**Table:**

*D*—H···*A*	*D*—H	H···*A*	*D*···*A*	*D*—H···*A*
C28—H28*B*···*Cg*1^i^	0.98	3.00	3.601 (7)	121
C5—H5···*Cg*2^ii^	0.93	2.89	3.693 (3)	145

Symmetry codes: (i) *x*+1, *y*, *z*; (ii) −*x*, −*y*, −*z*.
